# Supplementary material for: Associations between Particulate Matter and Otitis Media in Children: A Meta-Analysis
Source: Int J Environ Res Public Health. 2020 Jun 26;17(12):4604. doi: 10.3390/ijerph17124604 (PMC7345266; doi:10.3390/ijerph17124604)
Supplement: Supplementary file 1 [file ijerph-17-04604-s001.pdf]

Supplementary Tables

Supplementary Table A. Search strategy and result of each database. Titles or abstracts of records were retrieved.

Condition Number	Search Conditions	Number of Records		
		PubMed	Embase	Cochrane Library
#1	"Otitis Media"	22,043	25,153	2326
#2	"Particulate Matter" OR "Air Pollution" OR "Dust"	74,669	100,377	2574
#3	"Prevalence" OR "Incidence" OR "Morbidity" OR "Association" OR "Risk"	3,849,088	5,554,994	338,104
#4	#1 AND #2 AND #3	**41**	**52**	**3**

Supplementary Table B1. Newcastle - Ottawa quality assessment scale for case control studies and cohort studies. Except comparability, a point is allocated to each numbered item. Two points can be given for comparability.

Study type	Quality assessment scale
Case control study	**Selection**
	1) Is the case definition adequate?<br>a) yes, with independent validation (1)<br>b) yes, record linkage or based on self-reports (0)<br>c) no description (0)<br>2) Representativeness of the cases<br>a) consecutive or obviously representative series of cases (1)<br>b) potential for selection biases or not stated (0)<br>3) Selection of Controls<br>a) community controls (1)<br>b) hospital controls (0)<br>c) no description (0)<br>4) Definition of Controls<br>a) no history of otitis media (1)<br>b) no description of source (0)
	**Comparability**
	1) Comparability of cases and controls on the basis of the design or analysis<br>a) study controls for age (1)<br>b) study controls for any additional factor (1)
	**Exposure**
	1) Ascertainment of exposure<br>a) secure record (1)<br>b) structured interview where blind to case/control status (1)<br>c) interview not blinded to case/control status (0)<br>d) written self-report or medical record only (0)<br>e) no description (0)<br>2) Same method of ascertainment for cases and controls<br>a) yes (1)<br>b) no (0)<br>3) Non-Response rate<br>a) same rate for both groups (1)

	b) non respondents described (0)
	c) rate different and no designation (0)
Cohort study	**Selection**
	1) Representativeness of the exposed cohort
	a) truly representative of the average children in the community (1)
	b) somewhat representative of the average children in the community (1)
	c) selected group of users (0)
	d) no description of the derivation of the cohort (0)
	2) Selection of the non exposed cohort
	a) drawn from the same community as the exposed cohort (1)
	b) drawn from a different source (0)
	c) no description of the derivation of the non exposed cohort (0)
	3) Ascertainment of exposure
	a) secure record (1)
	b) structured interview (1)
	c) written self-report (0)
	d) no description (0)
	4) Demonstration that outcome of interest was not present at start of study
	a) yes (1)
	b) no (0)
	**Comparability**
	1) Comparability of cohorts on the basis of the design or analysis
	a) study controls for age (1)
	b) study controls for any additional factor (1)
	**Outcome**
	1) Assessment of outcome
	a) independent blind assessment (1)
	b) record linkage (1)
	c) self-report (0)
	d) no description (0)
	2) Was follow-up long enough for outcomes to occur
	a) yes (1)
	b) no (0)
	3) Adequacy of follow up of cohorts
	a) complete follow up - all subjects accounted for (1)
	b) subjects lost to follow up unlikely to introduce bias - small number lost → 70 % (1)
	c) follow up rate < 70 % and no description of those lost (0)
	d) no statement (0)

Supplementary Table B2. Quality assessment using the Newcastle–Ottawa Scale. After assessing the adequacy of case selection, comparability, and exposure, all articles were rated as having a low risk of bias.

Published	Authors	Study design	Q1	Q2	Q3	Q4	Q5	Q6	Q7	Q8	Quality
2006	Brauer M et al.	Cohort study	b	a	a	b	ab	c	a	b	7
2010	Zemek R et al.	Case-control study	a	a	b	b	ab	a	a	a	7
2011	MacIntyre EA et al.	Cohort study	b	a	a	b	ab	a	a	b	8
2014	MacIntyre EA et al.	Cohort study	a	a	a	a	ab	c	a	b	8
2016	Kousha T et al.	Case-control study	a	b	a	a	ab	a	a	a	8
2016	Strickland MJ et al.	Case-control study	a	b	b	a	ab	a	a	a	7
2016	Xiao Q et al.	Case-control study	a	b	b	a	ab	a	a	a	7
2016	Yao J et al.	Cohort study	a	a	a	b	ab	a	a	a	8
2017	Deng Q et al.	Cohort study	b	a	a	b	ab	c	a	b	7
2017	Girguis MS et al.	Case-control study	a	a	b	a	ab	a	a	a	8
2018	Girguis MS et al.	Case-control study	a	a	b	a	ab	a	a	a	8
2018	Park M et al.	Case-control study	a	a	a	b	ab	a	a	a	8
